# Environmental temperature and relative humidity shape post-emission aerosol fate and airborne influenza transmission

**DOI:** 10.1128/jvi.00634-26

**Published:** 2026-06-15

**Authors:** Xuan-Dung Nguyen, Bac Tran Le, Jacob Bleich, Constanza Espada, Wei Zhang, Xiu-Feng Wan

**Affiliations:** 1 NextGen Center for Influenza and Emerging Infectious Diseases, University of Missouri 14716, Columbia, Missouri, USA; 2 Department of Molecular Microbiology and Immunology, School of Medicine, University of Missouri 12271, Columbia, Missouri, USA; 3 Department of Electrical Engineering & Computer Science, College of Engineering, University of Missouri 199679, Columbia, Missouri, USA; 4 Bond Life Sciences Center, University of Missouri 14716, Columbia, Missouri, USA; Emory University School of Medicine, Atlanta, Georgia, USA

**Keywords:** temperature, relative humidity, airborne transmission, bioaerosol sampling, PathoSift Pro, particle size distribution, influenza A virus, swine model

## Abstract

Airborne transmission is a major route of influenza virus spread, yet how environmental conditions shape the persistence and downrange transport of infectious exhaled virions is not fully understood. Using a physiologically relevant swine model infected with A/California/04/2009 (H1N1), we investigated how temperature and relative humidity (T/RH) influence airborne influenza emission, persistence, and transmission under two environmental conditions: 20°C/50% RH (ambient indoor), and 7°C/73% RH (cold/high-humidity). Donor pigs shed comparable nasal viral loads across conditions, but naïve sentinel pigs housed 4 m away became infected 1 day earlier under ambient conditions. Breath and environmental air sampling showed that cold/high-humidity conditions transiently increased viral RNA in exhaled aerosols at 1 day post-infection (dpi), whereas ambient conditions supported greater and more persistent airborne viral burdens at 2–3 dpi, particularly at downrange locations. Controlled aerosol generation experiments further showed that ambient conditions enabled substantially greater recovery of infectious virus with distance, even though RNA-containing particles were transported under both T/RH states. Together, these results demonstrate that, under the tested environmental conditions, infectious influenza aerosols persisted longer and transmitted farther under the ambient indoor environment than in the cold/high-humidity environment. These findings establish that environmental temperature-humidity conditions shape post-emission aerosol fate, and thereby constrain the airborne transmission range of the influenza virus.

**IMPORTANCE:**

Influenza viruses spread efficiently through the air, yet the environmental conditions that determine whether exhaled virions remain infectious long enough to initiate new infections remain poorly defined. Using a swine model that closely replicates human expiratory aerosol output, we identify environmental temperature-humidity conditions as a critical determinant of airborne infectious range. Cold/high-humidity conditions increased early viral RNA levels near the host but failed to sustain infectious particles at a distance. In contrast, ambient conditions supported prolonged airborne suspension and rapid transmission to distant recipients. Controlled aerosolization experiments showed that infectious virus is transported far more effectively under ambient indoor conditions than in cold/high-humidity air despite similar RNA dispersal. These results reveal post-emission aerosol fate as the critical bottleneck in determining airborne influenza transmissibility. This mechanistic insight is essential for refining predictive models of influenza spread and developing environmental and public health strategies that more effectively limit airborne infection.

## INTRODUCTION

Influenza virus remains one of the most contagious human respiratory pathogens, causing annual epidemics despite large-scale vaccination efforts. Seasonal A(H1N1), A(H3N2), and influenza B viruses are estimated to cause approximately 36,000 deaths and 226,000 hospitalizations each year in the United States alone (1). Although vaccination coverage approaches ~40% of the population, current vaccines primarily reduce disease severity rather than fully preventing infection or onward transmission (2), and their effectiveness varies substantially across subtypes and seasons due to antigenic drift, pre-existing immunity, and vaccine mismatch (3–5). As a result, influenza continues to circulate efficiently, underscoring the need to understand the determinants of airborne spread, rather than focusing exclusively on disease attenuation.

Airborne transmission plays a central role in influenza epidemiology. Virus-laden respiratory particles are continuously generated during breathing, talking, coughing, and sneezing (6). Fine aerosols (≤5 µm) can remain suspended for hours and deposit in the lower respiratory tract, where infection initiates most efficiently (7, 8). Larger droplets (>5 µm) settle more rapidly and are more likely to deposit in the upper airway (8) but tend to retain moisture longer and maintain physicochemical conditions that are more favorable for viral stability, reflecting size-dependent aerodynamic and evaporation dynamics along a continuous particle spectrum (9). Despite recognition that both pathways contribute to transmission, the mechanisms that determine which pathway predominates under distinct environmental conditions remain unresolved.

Temperature and relative humidity (T/RH) strongly influence influenza infectious stability, aerosol behavior, and transmission efficiency (10–13). Low RH (~20–35%) and colder temperatures (e.g., 5°C) favor viral infectious stability and airborne persistence, whereas intermediate and high humidity accelerate infectivity loss, often producing non-linear outcomes that cannot be predicted solely from stability measurements (14). However, the degree to which environmental conditions influence airborne infectivity independent of donor shedding intensity has not been resolved, leaving uncertainty about how far virus-laden aerosols travel while remaining infectious. This gap has prevented translation of environmental observations into predictive models of influenza spread.

Human challenge studies and contact-tracing analyses have quantified viral shedding kinetics and secondary attack rates (15–17), with nasal virus titers typically peaking within 24–48 h of symptom onset (18, 19) and exhaled viral output often dominated by submicron aerosols (20–23). Yet, epidemiological data cannot manipulate environmental variables, limiting these observations to association rather than causation. Small-animal models, including mice, guinea pigs, hamsters, and ferrets, have been essential for studying influenza pathogenesis and transmission (24, 25). However, these models exhibit differences relative to human respiratory emission patterns. In particular, small-animal models generally produce lower total aerosol output than humans, likely reflecting differences in lung volume and ventilation rate (26), with measurable but relatively low aerosol emission reported in small-animal systems (27). Human respiratory aerosol generation has been well characterized, with measurable particle emission during tidal breathing and increased output during speech and other expiratory activities (21, 28). In addition, mice do not naturally transmit influenza, and particle-size distributions can vary across species and experimental systems (29). As a result, these systems, while indispensable for pathogenesis research, may not fully recapitulate human-like airborne emission dynamics.

To address this gap, we employed a swine model whose expiratory aerosol output closely mirrors that of humans (30, 31) and conducted controlled airborne virus sampling under distinct T/RH conditions. This combined infection and aerosolization system enabled us to disentangle donor viral shedding from post-emission environmental control of aerosol persistence and infectivity. We show that environmental temperature-humidity conditions are associated with whether exhaled virus remains confined to the near-source region or disperses downrange while retaining infectivity.

## RESULTS

### Nasal viral shedding was comparable across environmental conditions

**Fig 1:**
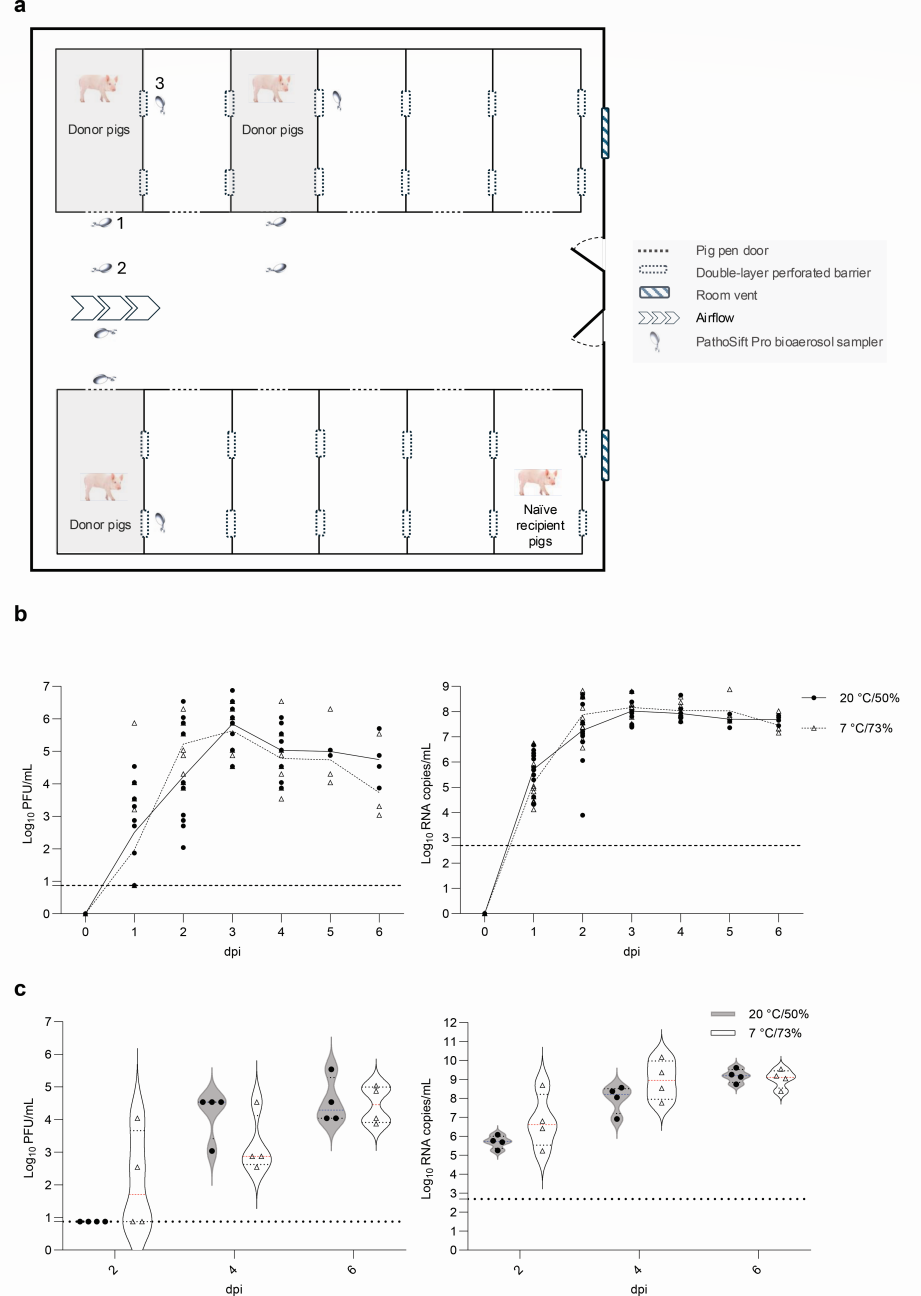
Experimental design and donor viral shedding under controlled environmental conditions. (**a**) Schematic of the swine airborne transmission experimental setup. Donor pigs were intranasally inoculated with A/California/04/2009 (H1N1) (CA/04-H1N1) and housed under either 20°C/50% relative humidity (RH) or 7°C/73% RH in climate-controlled, independently ventilated rooms. Naïve recipient pigs were housed 4 m from donors with no direct or indirect contact, ensuring airborne-only exposure. PathoSift Pro samplers were positioned at multiple locations, including adjacent to donor pens (front barred door panel) (position 1), at 0.5 m downrange (position 2), and adjacent to donor pens (side wire-mesh panel) (position 3), to capture breath and environmental air samples. Arrows indicate airflow direction within the room. (**b**) Infectious virus titers (left) and viral RNA copies (right) in nasal wash samples collected daily from donor pigs from 0 to 6 days post-infection (dpi) under both environmental conditions. (**c**) Bronchoalveolar lavage fluid (BALF) was collected from donor pigs infected with CA/04-H1N1 at 2, 4, and 6 days post-infection (dpi) under 20°C/50% RH and 7°C/73% RH. Infectious virus titers (left) and viral RNA copy numbers (right) were quantified by TCID_50_ assay and qRT-PCR, respectively. Each point represents an individual animal; lines indicate group means. Data were analyzed using linear mixed-effects models comparing temperature/RH conditions across dpi, followed by Tukey’s HSD test (*α* = 0.05). Dashed lines indicate limits of detection (0.872 log_10_ PFU/mL or 2.698 log_10_ RNA copies/mL).

To establish a baseline for donor viral burden before assessing airborne transport, we first quantified nasal shedding kinetics under two environmental conditions. Pigs (*n* = 12 per condition) were intranasally infected with A/California/04/09 (H1N1) (CA/04-H1N1) and housed at 20°C/50% RH (ambient/indoor simulation) and 7°C/73% RH, representing a colder, higher-humidity condition used to evaluate environmental effects on airborne virus persistence (Fig. 1a).

Viral titer became detectable in nasal washes by 1 day post-infection (dpi) in both groups and peaked at 3 dpi, with mean titers of 5.5 (±0.7) (mean ± standard deviation) and 5.8 (±0.7) log_10_ PFU/mL or 8.0 (±0.5) and 8.2 (±0.4) log_10_ RNA copies/mL under ambient and cold/high-humidity conditions, respectively (Fig. 1b). Although RNA copies at 2 dpi were modestly higher in the 7°C/73% RH group, neither peak titers nor overall shedding duration differed significantly between conditions (7.6 and 7.3 log_10_ RNA copies/mL). Viral loads declined thereafter, reaching 4–5 log_10_ PFU/mL or ~7 log_10_ RNA copies/mL by 6 dpi (Fig. 1b). Viral loads in bronchoalveolar lavage fluid (BALF) did not differ significantly between the two conditions (Fig. 1c), with infectious titers ranging from 0.9 to 4.5 and from 2.0 to 4.5 log_10_ PFU/mL, and viral RNA levels ranging from 5.7 to 9.2 and from 6.8 to 9.0 log_10_ RNA copies/mL, respectively.

These findings indicate that the two tested T/RH conditions did not substantially alter the magnitude or timing of upper-airway viral shedding, providing a baseline for evaluating environmental effects on airborne dispersal and transmission independent of donor replication intensity.

### Infected pigs predominantly exhale submicron aerosols and emit infectious influenza virus across aerosol and droplet sizes

**Fig 2:**
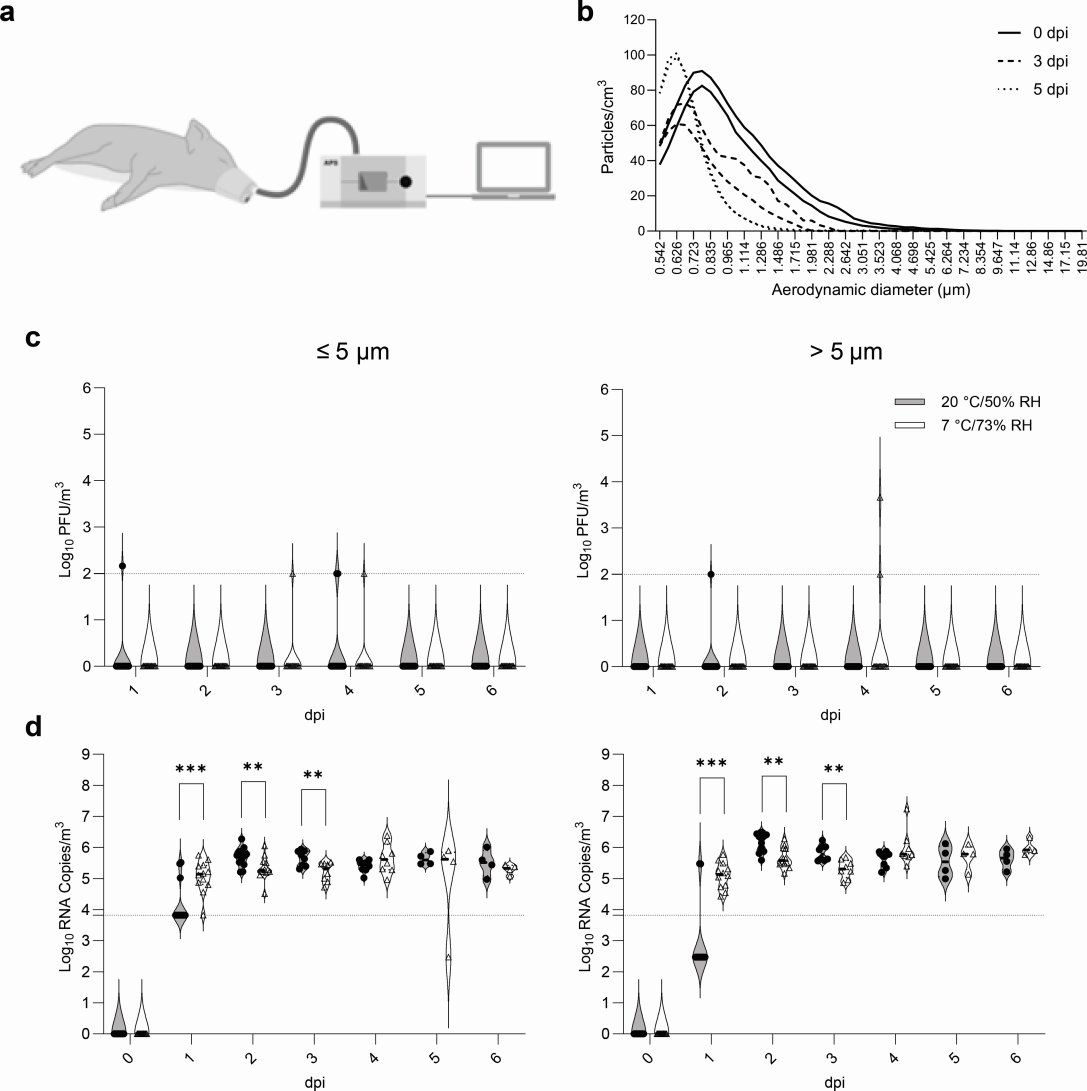
Exhaled particle size distributions and airborne influenza virus emission from infected pigs. (**a**) Schematic illustration of the pig mask and sampling configuration used to measure exhaled particle size distributions. (**b**) Aerodynamic particle size distributions of exhaled breath from pigs infected with CA/04-H1N1, measured using an Aerodynamic Particle Sizer (APS) at 0, 3, and 5 dpi. Curves represent mean particle concentrations across animals, demonstrating a unimodal distribution dominated by submicron particles within measurable range of sizer (0.54–20 µm). (**c**) Infectious virus titers and (**d**) viral RNA copies recovered from size-segregated exhaled particles collected using PathoSift Pro. Particles were classified as aerosols (≤5 µm) or droplets (>5 µm) and sampled longitudinally from 0 to 6 dpi under two environmental conditions (20°C/50% RH and 7°C/73% RH). Data points represent individual animals; lines indicate group means. For aerosols, two infected pigs shed detectable infectious virus under 20°C/50% RH (one at 1 dpi and one at 4 dpi), while two infected pigs shed infectious virus under 7°C/73% RH (one at 3 dpi and one at 4 dpi). For droplets, only one pig shed infectious virus at 2 dpi under 20°C/50% RH, whereas two pigs shed infectious virus at 4 dpi under 7°C/73% RH. Statistical comparisons between environmental conditions across dpi were performed using linear mixed-effects models followed by Tukey’s HSD test (*α* = 0.05). Dashed lines indicate limits of detection (1.997 log_10_ PFU/m^3^ or 3.823 log_10_ RNA copies/m^3^). Asterisks indicate statistical significance (*, *P* < 0.05; **, *P* < 0.01; ***, *P* < 0.001; and ****, *P* < 0.0001).

To define the characteristics of airborne virus emission from donors, exhaled particle size distributions were quantified using an aerodynamic particle sizer (Fig. 2a) (APS Model 3321; TSI, Shoreview, MN, USA). Across 0, 3, and 5 dpi, pigs under anesthesia exhaled 10^5^–10^8^ particles/m³ (0.54–20 μm), with 99.81% ≤5  µm and a single peak below 1 μm (Fig. 2b).

Breath was sampled directly from anesthetized pigs through size-segregated sampling with PathoSift Pro (31). Characterization experiments using fluorescent polystyrene latex (PSL) beads confirmed that small particles were largely transmitted through the 5 µm mesh, whereas larger particles were preferentially retained (Fig. S1).

Recovery of infectious virus from breath samples occurred intermittently and was detected in a subset of animals at specific time points (Fig. 2c), consistent with the low abundance and stochastic detection of viable virus in exhaled aerosols. For aerosols, two infected pigs shed detectable infectious virus under 20°C/50% RH (one at 1 dpi and one at 4 dpi), while two infected pigs shed infectious virus under 7°C/73% RH (one at 3 dpi and one at 4 dpi). For droplets, only one pig shed infectious virus at 2 dpi under 20°C/50% RH, whereas two pigs shed infectious virus at 4 dpi under 7°C/73% RH.

At RNA level, PathoSift Pro recovered viral RNA in both aerosols (≤5  µm) and droplets (>5  µm) (Fig. 2d). Droplet fractions contained the highest viral RNA levels (6.1 [±0.3] log_10_ RNA copies/m^3^ at 20°C/50% RH and 5.7 [±0.3] log_10_ RNA copies/m^3^ at 7°C/73% RH), whereas aerosols carried slightly lower levels (5.7 [±0.3] and 5.3 [±0.4] log_10_ RNA copies/m^3^, respectively).

Although pigs under 7°C/73% RH released more RNA on 1 dpi, airborne viral burdens were higher under 20°C/50% RH on 2 and 3 dpi, after which differences between conditions diminished. Importantly, infectious virus was present across the full aerodynamic range, confirming that donors simultaneously emit particles capable of near-field deposition (>5 µm) and prolonged airborne suspension (≤5 µm).

Together, these data show that infected pigs rapidly exhale virus-laden particles across the full transmissible size spectrum, and that both aerosols and droplets serve as infectious vehicles early in infection, enabling short- and long-range airborne spread.

### Environmental temperature-humidity conditions shape near-field accumulation versus far-field dispersion of airborne virus

To determine how environmental conditions influence airborne virus distribution beyond host emission, viral RNA was quantified in environmental air samples collected at defined spatial locations under 7°C/73% RH and 20°C/50% RH. Three sampling positions were evaluated: (i) immediately adjacent to donor pens at the front barred door panel, (ii) 0.5 m downrange at the same panel, and (iii) adjacent to donor pens at the side wire-mesh panel (Fig. 1a).

**Fig 3:**
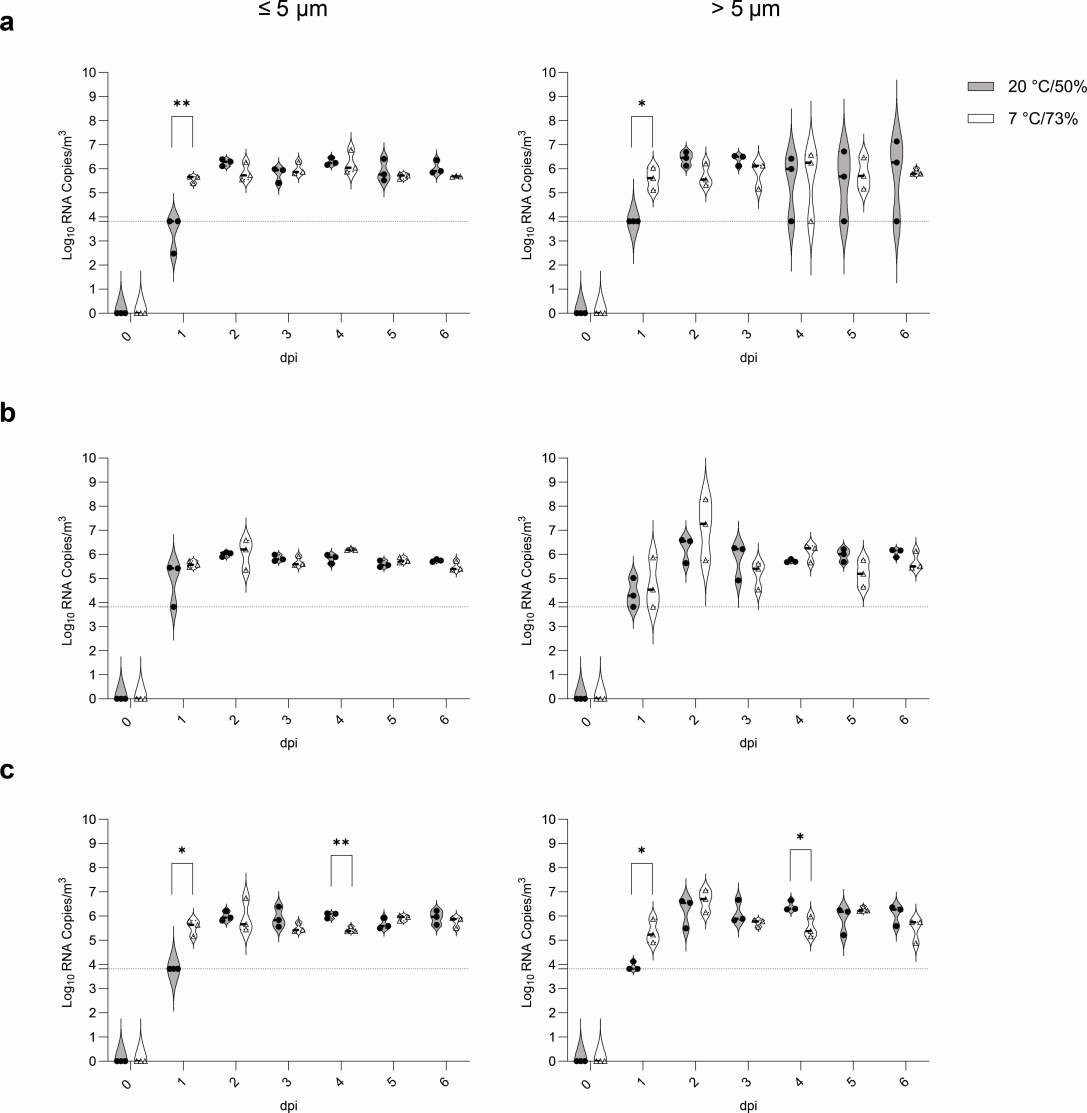
Environmental temperature-humidity conditions influence near-field accumulation and downrange detection of airborne viral RNA. Viral RNA in aerosols (≤5 µm) and droplets (>5 µm) was quantified from air samples collected around pigs infected with CA/04-H1N1 under two environmental conditions: 20°C/50% RH (ambient) and 7°C/73% RH (cold/high-humidity). (**a**) Samples collected adjacent to donor pens (near field; front barred door panel), (**b**) samples collected 0.5 m downrange from the same panel, and (**c**) samples collected adjacent to donor pens (side wire-mesh panel). Data points represent individual samples; horizontal lines indicate group means. Viral RNA levels were compared between environmental conditions across days post-infection using two-way repeated-measures ANOVA, followed by Tukey’s HSD test (*α* = 0.05). Dashed horizontal lines indicate the limit of detection (3.823 log_10_ RNA copies/m^3^). Asterisks indicate statistical significance (*, *P* < 0.05; **, *P* < 0.01; ***, *P* < 0.001; and ****, *P* < 0.0001).

Near-field samplers positioned adjacent to donor pens (front barred door panel and side wire-mesh panel) reflected breath-associated viral dynamics over the course of infection. At 1 dpi, viral RNA levels at the front barred door panel were significantly higher under cold/high-humidity conditions, with aerosol and droplet concentrations of 5.6 (±0.2) and 5.6 (±0.5) log_10_ RNA copies/m^3^, respectively, compared to 3.3 (±0.8) and 3.8 (±0.0) log_10_ RNA copies/m^3^ under ambient conditions. A similar pattern was observed at the side wire-mesh panel, where aerosol and droplet viral RNA levels reached 5.5 (±0.3) and 5.3 (±0.5) log_10_ RNA copies/m^3^ under cold/high-humidity conditions, versus 3.8 (±0.0) and 3.9 (±0.2) log_10_ RNA copies/m^3^ under ambient conditions. However, this early enhancement did not persist. From 2 to 6 dpi, near-field viral burdens converged across conditions, with only sporadic differences observed (Fig. 3). Notably, the initial differences in viral RNA levels between environmental conditions diminished at 0.5 m downrange, indicating that RNA-containing particles were transported under both conditions, with relatively limited differences at distance.

**Fig 4:**
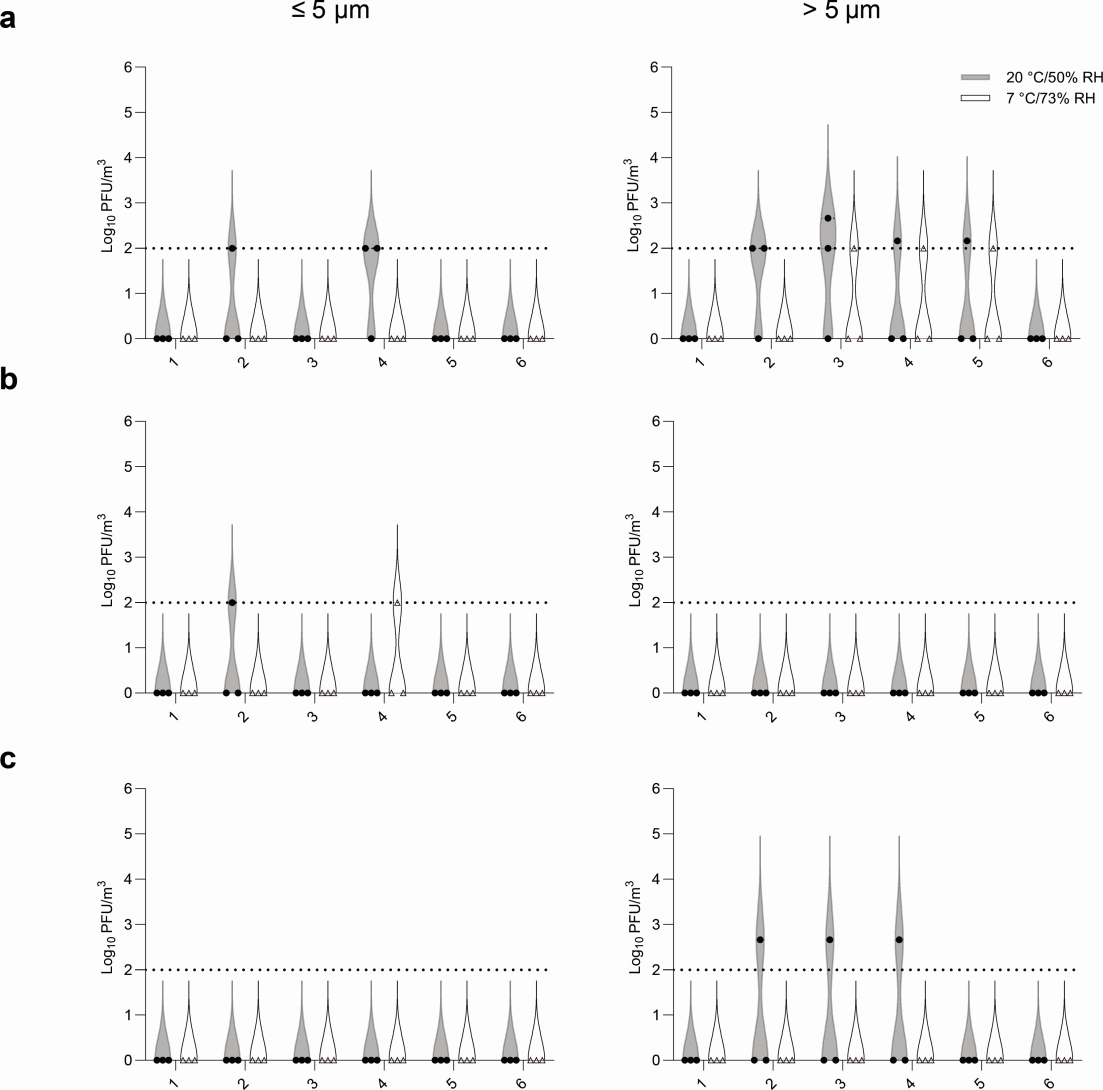
Recovery of infectious airborne influenza virus at multiple spatial locations under different environmental temperature-humidity conditions. Infectious A/California/04/2009 (H1N1) (CA/04-H1N1) virus titers were measured in size-segregated air samples collected using PathoSift Pro from donor rooms under 20°C/50% RH and 7°C/73% RH. Samples were collected at defined locations relative to donor pigs, including: (**a**) adjacent to the donor pen (front barred door panel), where infectious virus was detected in three aerosol (≤5 µm) and six droplet (> 5 µm) samples under ambient conditions, and three droplet samples under cold/high-humidity conditions; (**b**) 0.5 m downrange, where infectious virus was detected in a single aerosol sample under each condition; and (**c**) adjacent to donor pens (side wire-mesh panel), where infectious virus was detected in three droplet samples, all under ambient conditions. Individual symbols represent independent sampling events across days post-infection, with lines connecting samples collected on the same day. Dashed horizontal lines indicate the limit of detection (1.997 log_10_ PFU/m^3^). Infectious virus was recovered more frequently and at greater distances under ambient conditions, whereas recovery was sporadic and largely confined to near-source locations under cold/high-humidity conditions.

Infectious virus recovery showed a distinct pattern. Infectious virus was recovered predominantly under ambient conditions and was rare under cold/high-humidity conditions (Fig. 4). The highest recovery occurred adjacent to donor pens (front barred door panel) under 20°C/50% RH, where infectious virus was detected in 3 of 18 aerosol (≤5 µm) samples and 6 of 18 droplet (>5 µm) samples (Fig. 4a). At the same position under 7°C/73% RH, no infectious virus was detected in aerosol samples, and only 3 of 18 droplet samples were positive. Detection decreased with distance, with only a single positive aerosol sample identified at 0.5 m downrange (front barred door panel) across 18 aerosol and 18 droplet samples under each condition (Fig. 4b). At the positions adjacent to donor pens (side wire-mesh panel), only 3 of 18 aerosol samples were positive under 20°C/50% RH, whereas none were positive under 7°C/73% RH; no droplet samples were positive under either condition (Fig. 4c).

Together, these findings demonstrate that viral RNA is transported beyond the near field under both environmental conditions, with minimal differences in RNA detection at distance. In contrast, recovery of infectious virus is strongly environment dependent. Ambient conditions support detectable viable virus in the near field and, to a limited extent, downrange, whereas cold/high-humidity conditions promote near-field RNA accumulation but markedly reduce infectious virus recovery across all positions. These results indicate that environmental conditions primarily influence viral viability rather than the physical dispersal of virus-containing particles.

### Environmental conditions modulate long-range airborne influenza transmission

To evaluate whether environmental conditions translate into altered transmission efficiency, sentinel pigs were housed 4 m away from infected donors under 20°C/50% RH or 7°C/73% RH (Fig. 1a). Despite similar nasal viral shedding kinetics between donor groups, transmission outcomes diverged markedly.

**Fig 5:**
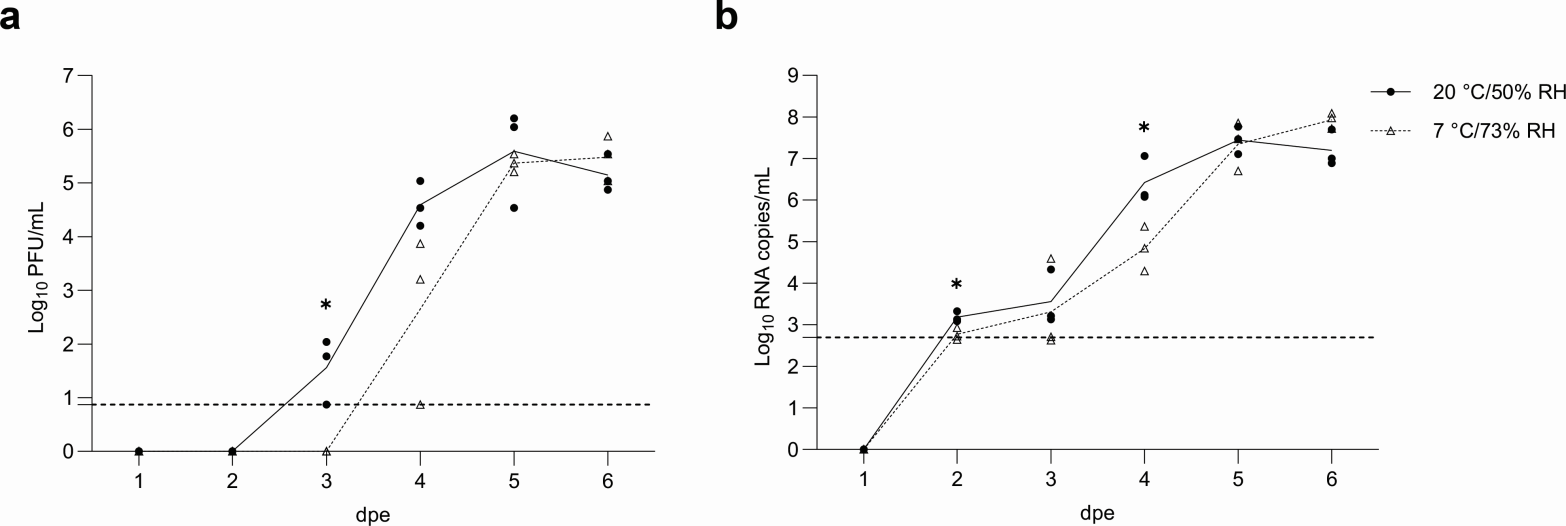
Environmental temperature-humidity conditions modulate airborne transmission to recipient pigs. Naïve sentinel pigs (*n* = 3 per condition) were housed 4 m from donor pigs infected with CA/04-H1N1 under two environmental conditions: 20°C/50% RH (ambient) and 7°C/73% RH (cold/high-humidity). (**a**) Recovery of infectious virus (log_10_ PFU/mL) and (**b**) viral RNA levels (log_10_ RNA copies/mL) in nasal wash samples collected daily from 1 to 6 days post-exposure (dpe). Points represent individual animals; lines indicate group means. Transmission occurred earlier and more consistently under ambient conditions, whereas infection was delayed and attenuated under cold/high-humidity conditions. Data were analyzed using two-way repeated-measures ANOVA with day post-exposure and T/RH condition as fixed effects, followed by Tukey’s HSD test (*α* = 0.05). Dashed lines indicate limits of detection (0.872 log_10_ PFU/mL or 2.698 log_10_ RNA copies/mL). Asterisks indicate statistical significance (*, *P* < 0.05; **, *P* < 0.01; ***, *P* < 0.001; and ****, *P* < 0.0001).

Infectious virus was detected in all three sentinel pigs by 3 days post-exposure (dpe) under 20°C/50% RH, whereas no sentinel showed infectious virus until 4 dpe under 7°C/73% RH (Fig. 5a). Viral RNA mirrored this delay: RNA levels were significantly higher under ambient conditions on 2 and 4 dpe (*P* < 0.01), while titers remained low and transient in the cold/high-humidity room (Fig. 5b). Two-way repeated-measures ANOVA confirmed strong effects of exposure day (*P* < 0.001), T/RH conditions (*P* = 0.034), and their interaction (*P* = 0.011), indicating that environmental conditions modulated both timing and magnitude of airborne infection.

These differences were not explained by donor shedding: although 7°C/73% RH enhanced near-field RNA at 1 dpi, only 20°C/50% RH supported recovery of infectious virus at downrange locations (Fig. 3). The earlier and more consistent infection of sentinels under ambient conditions therefore reflects enhanced airborne persistence and dispersal, not only increased donor viral burden.

### Ambient T/RH preserves airborne influenza infectivity, whereas cold/high-humidity conditions accelerate viability loss

**Fig 6:**
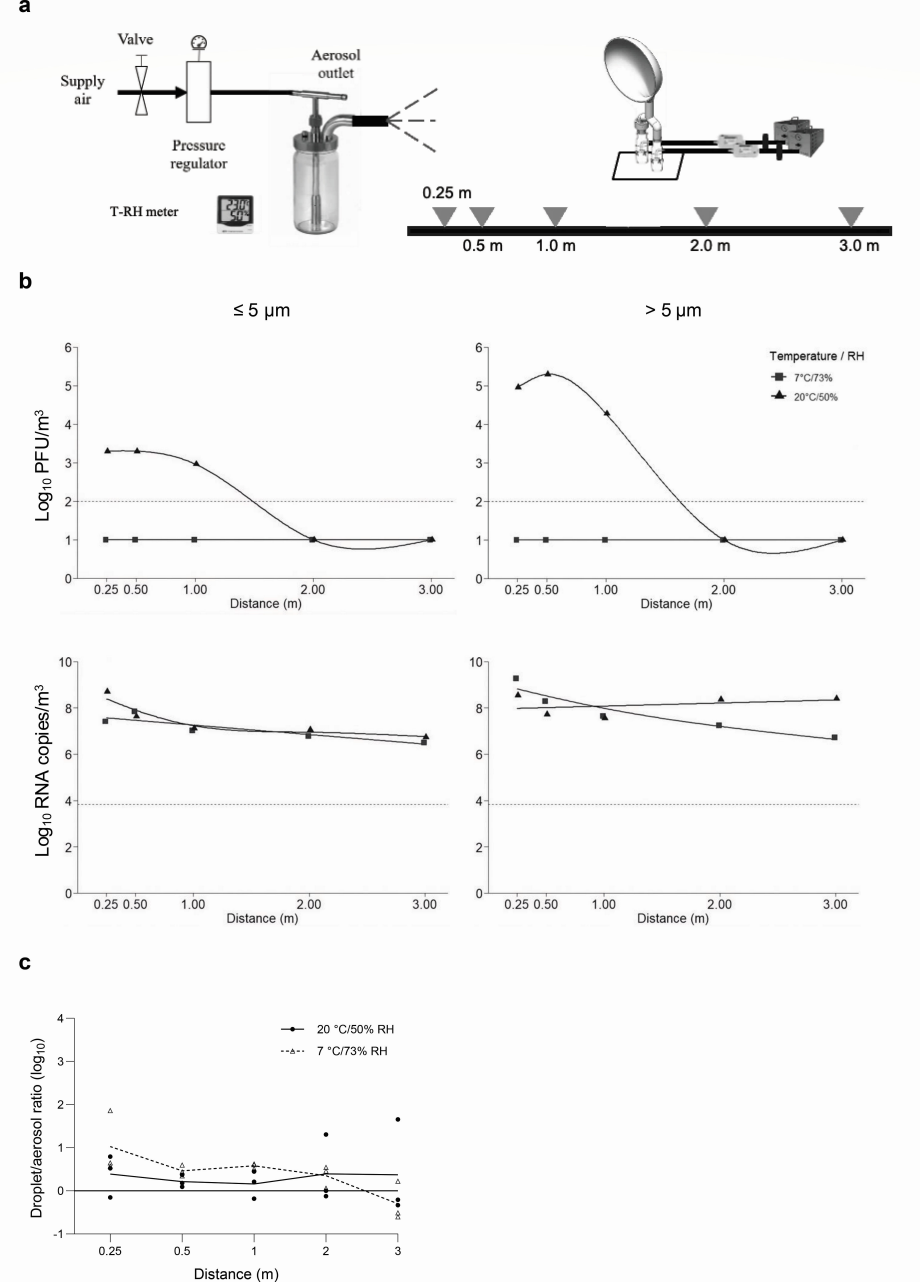
Environmental temperature-humidity control of airborne influenza infectivity during aerosol transport. (**a**) Schematic of the controlled aerosol transport experiment. CA/04-H1N1 virus was aerosolized and sampled at increasing distances (0.25, 0.5, 1, 2, and 3 m) under two T/RH conditions: 20°C/50% RH (ambient) and 7°C/73% RH (cold/high-humidity). Size-segregated virus-laden particles were collected using PathoSift Pro to distinguish aerosols (≤5 µm) from droplets (>5 µm). (**b**) Generalized additive model (GAM) fits showing distance-dependent recovery of infectious virus (PFU/m^3^) and viral RNA levels (log10 RNA copies/m^3^) in aerosols (≤5 µm) and droplets (>5 µm) under each T/RH condition. (**c**) Ratio of droplet-associated to aerosol-associated viral RNA at different distances under two environmental conditions (20°C/50% RH and 7°C/73% RH). Points represent experimental measurements; curves indicate GAM-modeled trends. Dashed horizontal lines denote limits of detection (1.997 log_10_ PFU/m^3^ or 3.823 log_10_ RNA copies/m^3^). The data used in the GAM models are available in Fig. S3.

To assess whether environmental conditions directly affect airborne virus survival and transport independent of the host, CA/04-H1N1 was aerosolized and sampled at graded distances under 20°C/50% RH and 7°C/73% RH (Fig. 6a).

Under ambient conditions (20°C/50% RH), viral RNA was detected in both aerosols (≤5 µm) and droplets (>5 µm) at all sampled distances up to 3 m, indicating efficient physical transport of virus-laden particles (Fig. 6b). RNA levels, particularly in droplet fractions, showed minimal change with increasing distance; 1–2 log reduction at 3 m position (Fig. 6b; Fig. S3). In contrast to RNA persistence, the infectious virus exhibited distance-dependent loss of viability under ambient conditions. Infectious virus was consistently recovered from both aerosol and droplet fractions at short distances (≤1 m), with titers declining progressively with distance and being undetectable at 2–3 m.

When environmental conditions were shifted to cold/high-humidity air (7°C/73% RH), viral RNA remained detectable across the full 3 m distance range in both aerosols and droplets, indicating sustained particle transport (Fig. 6b). However, recovery of infectious virus was markedly reduced and was rare or absent for both aerosol and droplet fractions even at the shortest sampling distance (0.25 m).

Analysis of the relative distribution of viral RNA between droplets and aerosols revealed distinct distance-dependent patterns across environments (Fig. 6c). Under ambient conditions (20°C/50% RH), the droplet-to-aerosol RNA ratio remained relatively stable across distances, ranging from 0.39 (±0.49) to 0.37 (±0.09). In contrast, under cold/high-humidity conditions (7°C/73% RH), the droplet-to-aerosol RNA ratio decreased with increasing distance, declining from 1.03 (±0.72) at 0.25 m to 0.06 (±0.50) at 1 m; by 3 m, aerosol-associated RNA exceeded droplet-associated RNA (0.29 ± 0.45).

Statistical analysis supported these observations. Two-way repeated-measures ANOVA identified significant effects of sampling distance (*P* = 0.0039), T/RH condition (*P* = 0.004), and their interaction (*P* < 0.0001) on droplet-associated infectivity. Across both environmental conditions, droplet fractions contained higher infectious titers than aerosol fractions; however, sustained recovery of infectious virus with increasing distance was observed only under ambient T/RH conditions.

Together, these results demonstrate that while viral RNA is transported across distances under both environmental conditions, recovery of infectious virus is strongly influenced by T/RH, with cold/high-humidity conditions associated with more rapid loss of airborne infectivity.

## DISCUSSION

In this study, we examined how environmental temperature-humidity conditions shape influenza airborne fate, viability, and transmission using a swine model. Despite comparable nasal shedding across environmental conditions, airborne transmission outcomes diverged markedly: ambient conditions (20°C/50% RH) supported prolonged virion suspension, downrange dispersal, and earlier infection of sentinel pigs, whereas cold/high-humidity environment (7°C/73% RH) delayed infection despite similar donor viral burden. Controlled aerosolization experiments provided mechanistic insight into this divergence: the tested cold/high-humidity condition did not inhibit viral emission but instead promoted rapid loss of airborne infectivity despite RNA detection to at least 3 m. Thus, environmental temperature-humidity conditions, rather than donor shedding magnitude alone, were a primary determinant of the persistence of infectious airborne virus under the tested conditions. Together, our findings support a mechanistic model in which environmental T/RH conditions shape post-emission aerosol fate and airborne infectivity.

In our study, temperature and relative humidity covaried between the two tested environments, so the independent effects of temperature versus humidity cannot be fully separated. Future factorial studies will be needed to fully disentangle independent T versus RH effects. Nevertheless, the observed divergence in airborne infectivity under cold/high-humidity versus ambient conditions is consistent with prior work demonstrating that environmental temperature–humidity conditions strongly influence aerosol persistence and virus stability (11, 13, 32).

The particle size distribution of exhaled breath from pigs, measured under anesthesia and therefore reflecting tidal breathing, was characterized by a strong dominance of fine particles with a substantial submicron fraction within the APS detection range (0.54–20 µm). This pattern is consistent with the fine-particle component reported in human quiet breathing, although human respiratory emissions arise from multiple physiological activities (e.g., speaking, coughing, and sneezing) that generate broader, multimodal distributions (20–23). Accordingly, these measurements capture a defined component of respiratory aerosol generation rather than the full spectrum of expiratory particles. Within this constrained measurement framework, prior human studies have similarly reported that particles generated during normal breathing are enriched in the submicron and small-particle range, with larger particle modes emerging when wider size ranges and additional respiratory activities are included; differences across studies and species likely reflect biological, behavioral, and methodological factors. Quantitatively, pigs exhaled 1,185 (±152) particles/cm^3^, comparable to concentrations reported for human tidal breathing (22), supporting their use for evaluating both near- and far-field airborne transmission under physiologically relevant aerosol output. In contrast, small animal models (e.g., ferrets, guinea pigs, and hamsters) are expected to produce lower absolute aerosol outputs due to substantially smaller respiratory volumes and airflow, and may exhibit distinct size distribution profiles (26, 27), which may influence airborne transmission dynamics across experimental systems. Collectively, these findings support the pig as a physiologically relevant model for investigating environmentally modulated airborne influenza transmission.

Environmental T/RH conditions influenced the spatial distribution of airborne virus, but their effects differed for viral RNA and infectious virus. Under cold/high-humidity conditions (7°C/73% RH), viral RNA levels in breath and near-cage air were higher at 1 dpi than under ambient conditions (20°C/50% RH), consistent with greater early near-source accumulation. However, viral RNA was detected at 0.5 m under both conditions, and differences between environments were less pronounced at this distance, indicating that RNA-containing particles were transported beyond the immediate release zone in both settings. Thus, the primary environmental effect on viral RNA distribution was most evident in the near field, whereas differences in downrange recovery were substantially more pronounced for infectious virus than for RNA.

The results of airborne transmission to recipient pigs mirror these distribution differences. Under 20°C/50% RH, all sentinel pigs shed RNA copies by 2 dpe and infectious virus by 3 dpe in nasal wash samples. In contrast, under 7°C/73% RH, infection was delayed until 4 dpe, despite comparable early donor shedding. This pattern aligns with prior guinea pig studies demonstrating T/RH-dependent reductions in influenza transmission (11). These results suggest that the tested cold/high-humidity condition limited the airborne persistence of infectious virus despite conditions that may, in some systems, support virus stability (13).

Aerosol experiments further supported a mechanistic explanation for the observed environmental effects on airborne infectivity. Under cold/high-humidity conditions (7°C/73% RH), the infectious virus was not maintained over distance, whereas under ambient conditions (20°C/50% RH), the infectious virus was consistently recovered. Viable CA/04-H1N1 was recovered up to 1–2 m away under ambient conditions, whereas no infectious virus was detected at distance under cold/high-humidity conditions despite persistent detection of viral RNA (Fig. 6). Prior work has demonstrated that the relationship between RH and influenza virus stability is complex, non-monotonic, and dependent on aerosol composition and experimental system. Early aerosol studies established that RH exerts a strong influence on influenza virus survival, often greater than that of temperature, with substantial variation in infectivity observed across RH conditions (33–35). Subsequent studies further indicate that influenza virus stability frequently exhibits regime-dependent behavior, with reduced infectivity often observed at intermediate RH, while infectivity may be partially preserved at both low RH, following solute crystallization, and at high RH depending on droplet composition (32, 36). More recent studies further highlight interactions between RH and aerosol composition in modulating viral infectivity (14). Consistent with this, transmission studies have shown that influenza virus spread is highly sensitive to RH and temperature (10–12), whereas experiments using physiologically relevant airway surface liquid demonstrate that RH-dependent decay can be substantially attenuated, with infectivity retained across a broad humidity range (13). Together, these findings indicate that high RH does not uniformly promote influenza virus inactivation; however, some experimental systems, including human cough aerosol models, have reported reduced airborne infectivity at higher RH (37).

In this context, our findings do not contradict prior reports of preserved infectivity at high RH, but instead highlight that airborne virus fate reflects the combined effects of virion stability and aerosol transport dynamics. At elevated RH, respiratory droplets retain more water and equilibrate at larger diameters, which promotes more rapid gravitational settling and reduces airborne residence time, thereby limiting long-range transport of infectious particles (32, 36, 38). Thus, even in scenarios where high RH may preserve virion infectivity in suspended droplets, our data suggest that under cold/high-humidity conditions, aerodynamic constraints likely dominate, preventing infectious virus from remaining airborne long enough to be recovered at distance. This interpretation is supported by the continued detection of viral RNA despite the absence of recoverable infectious virus, consistent with physical transport of viral material but inefficient maintenance of infectious airborne particles (Fig. 6). Collectively, our results extend prior work by demonstrating that, in a swine aerosol transmission setting, environmental humidity influences not only intrinsic viral stability but also the size-dependent physical processes governing aerosol suspension and transport. By explicitly measuring infectious virus over distance, our study provides a transmission-relevant framework that integrates these processes. This distinction is important because most prior studies evaluate viral stability in static aerosol systems, whereas our approach captures the combined effects of aerosol transport and viral persistence. Under the conditions tested here, these physical constraints appear to outweigh any potential preservation of infectivity at high RH. Whether cold, high humidity additionally contributes to direct virion inactivation, independent of particle fate, remains an open question and warrants further investigation.

Overall, this work establishes environmentally mediated post-emission aerosol fate as a key determinant of airborne infectious range in a physiologically relevant emitter system. Under ambient temperature and moderate humidity, virions undergo evaporative reduction and remain suspended at downrange locations, whereas cold temperature and elevated humidity favored retention of larger hydrated droplets and reduced airborne persistence of infectious virus and limited recovery at distance. Thus, environmental temperature-humidity conditions constrained the transition between greater near-field accumulation and downrange airborne persistence of infectious virus.

This study evaluated two discrete T/RH conditions using a single influenza A(H1N1) virus strain. Although these conditions reflect commonly encountered indoor and winter environments, additional humidity and temperature ranges may further delineate inflection points for airborne infectivity. Particle size distributions in exhaled breath were characterized using an APS; however, environmental particle size distributions at increasing distances from the source were not measured and may provide additional insight into humidity-dependent particle transport dynamics. Infectious recovery relied on TCID_50_ assays, which may underestimate low-abundance infectious aerosol fractions; however, transmission divergence across T/RH states was consistent across virological endpoints. Only immunologically naïve swine were studied, and pre-existing immunity may alter shedding kinetics and airborne viral load in field settings. Finally, a single airflow regime was used, and future work should examine additional airflow regimes, strengths, and directions.

In summary, environmental temperature-humidity conditions shape airborne influenza virus transmission under conditions where donor shedding magnitude is comparable. Ambient conditions supported persistence of infectious airborne virus and effective transmission, whereas cold/high-humidity conditions limited recovery of infectious virus at distance despite detectable viral RNA. These findings indicate that post-emission aerosol fate, shaped by environmental temperature-humidity conditions, influences whether exhaled virus remains airborne and infectious over distance.

## MATERIALS AND METHODS

### Viruses and cell lines

Influenza A human isolate, A/California/04/2009 (H1N1) (CA/04-H1N1), was used in this study. The Madin-Darby canine kidney (MDCK) SIAT1 cells kindly provided by Dr. Mikhail Matrosovich (39) were used for virus propagation. Virus stocks were passaged once in MDCK-SIAT1 cells, aliquoted, and stored at −80°C until use in aerosol sampling and animal studies.

MDCK-SIAT1 cells were maintained in Dulbecco’s modified Eagle medium (11965-084; Life Technologies Corporation, Grand Island, New York, USA), supplemented with 10% fetal bovine serum (A52568-01; Life Technologies Corporation) and penicillin/streptomycin (R15140-122; Life Technologies Corporation) at 100 μg/mL. For viral propagation, the cell was cultured in Opti-MEM I (11058-021 reduced serum medium; Life Technologies Corporation) supplemented with penicillin/streptomycin (15140-122; Life Technologies Corporation) at 100 μg/mL and TPCK-trypsin (T1426; St. Louis, MO, USA) at 1 μg/mL.

### Hemagglutination (HA) and hemagglutination inhibition (HAI) assays

The HA and HAI assays were performed using 0.5% turkey erythrocytes as described elsewhere (Ref 7209403; Lampire Biological Laboratory, Pipersville, PA, USA).

### 50% Tissue culture infectious dose (TCID_50_)

For viral titration, TCID_50_ was determined on MDCK cells. Briefly, cells were seeded in a 96-well plate at a density of 2 × 10^4^ cells per well in Opti-MEM I (11058-021 reduced serum medium; Life Technologies Corporation) and incubated at 37°C with 5% CO_2_ for 20 h. The test sample was serially diluted 10-fold in Opti-MEM I (11058-021 reduced serum medium; Life Technologies Corporation) supplemented with 1 µg/mL TPCK-trypsin (T1426; St. Louis, MO, USA). After removing the medium and washing the plates three times with phosphate-buffered saline (PBS), 200 μL of each dilution was added to each well in quadruplicate. Plates were incubated at 37°C with 5% CO_2_ for 72 h, and cytopathic effects were monitored daily by microscopy. The supernatants were collected and tested using a hemagglutination (HA) assay with 0.5% erythrocytes. The number of positive and negative wells for each dilution was recorded. TCID_50_ values were calculated using the Reed and Muench method (40, 41) and converted to PFU using the approximation 1 TCID_50_ = 0.7 PFU.

### RNA extraction and quantitative RT-PCR (qRT-PCR)

Viral RNA was extracted using a GeneJET Viral DNA/RNA extraction kit (K0821; Thermo Scientific, Waltham, MA, USA) according to the manufacturer’s instructions. RNA samples were processed for quantification using TaqMan Fast Virus 1-Step qRT-PCR (4444434; Thermo Fisher Scientific, Waltham, MA, USA), with M gene-specific primers 5′-GACCRATCCTGTCACCTCTGAC-3′ (forward primer), 5′-AGGGCATTYTGGACAAAKCGTCTA-3′ (reverse primer) (Invitrogen, Waltham, MA, USA), and 6-carboxyfluorescein (FAM) labeled probe 5′-TGCAGTCCTCGCTCACTGGGCACG-3′ (Eurofins Genomics, Louisville, KY, USA). The viral RNA copies were determined using a standard curve generated from a plasmid containing the M gene of A/Puerto Rico/8/1934 (H1N1) (42). The copy number determination by qRT-PCR was performed in triplicate.

### Aerosol sampling with PathoSift Pro

Virus-laden aerosol particles were collected using PathoSift Pro (31), a size-selective bioaerosol sampler. The device features a 20 cm conical inlet coupled to two BioSampler bottles (BioSampler 5 mL, Cat 225-9593; SKC, Eighty-Four, PA, USA), each operated at 12.5 L/min, as monitored by TSI flowmeter (Ref. 5200-2; TSI). A 5 µm stainless steel filter (C0000673510; 316L stainless steel, Artesian Systems, McKinnon, WY, USA) was installed in one channel to segregate aerosols (≤5 µm) from droplets (>5 µm), while the second channel collected total particles. These size categories represent operational particle fractions defined by the sampler configuration, recognizing that respiratory particles exist along a continuous size distribution. Particles were collected into sampling media (Opti-MEM supplemented with penicillin/streptomycin 100 μg/mL), and collection bottles were maintained on ice.

To characterize particle transmission across the 5 µm stainless steel mesh used in the PathoSift Pro system, a validation experiment was performed using PSL beads (43, 44) of defined diameters (1, 2, and 10 µm, Thermo Fisher Scientific, Cat #F8816, F8826, and F8836; 5 µm, CD Bioparticles, DCFG-L011). PSL suspensions were aerosolized and introduced into the sampler under the same airflow conditions used in the transmission experiments. The PathoSift Pro was operated with the mesh installed in one sampling channel and without the mesh in the parallel channel, consistent with the configuration used for aerosol sampling experiments. Aerosol particles were collected in the BioSampler bottles connected to each channel, and particle recovery was quantified for each bead size using flow cytometry (Cytek Aurora full spectrum analyzer, CytekBio, Fremont, CA, USA). Ten thousand events were acquired, and equal density of events was determined by plotting SSC versus time. The relative particle recovery between the mesh channel and the total-particle channel was used to evaluate size-dependent particle transmission across the mesh. Each experiment was performed in triplicate.

Data show that approximately 88% of 1 µm particles were transmitted through the mesh, whereas larger particles showed progressively lower transmission, with 10 µm particles effectively retained by the mesh (Fig. S1). These measurements were used to define the operational size-separation characteristics of the PathoSift Pro system.

Sampling durations were 30 min (breath), 60 min (environmental air), and 15 min (aerosolization experiments). Samples were analyzed for viral RNA and infectivity as previously described (31). Each experiment was performed in triplicate.

### Animal experiment

All work was performed in climate-controlled large-animal ABSL-2 rooms within the NextGen Center for Influenza and Emerging Infectious Diseases under independent HVAC systems with no recirculation of airflow. Two environmental conditions were used: 20°C/50% RH, approximating typical climate-controlled indoor environments, and 7°C/73% RH, representing a colder, higher-humidity condition (e.g., winter outdoor environments) selected to evaluate environmental effects on airborne virus persistence. Each room remained physically isolated with dedicated personnel access to prevent cross-exposure between environmental settings. The animal rooms were maintained at 15 air changes per hour under standard facility ventilation conditions.

#### Donor animals and inoculation

Twenty-four pigs were randomly assigned to the two experimental rooms (*n* = 12 per condition) and housed in three pens per room (four pigs per pen). Donor animals were anesthetized with Telazol–Xylazine (3.0 and 1.0 mg/kg intramuscularly, respectively) and intranasally inoculated with 10^6^ TCID_50_ of CA/04-H1N1 in 2 mL, evenly divided between each nostril. For breath aerosol sampling, anesthesia was increased to 5.0 mg/kg Telazol (Zoetis, 10004135) and 2.0 mg/kg Xylazine (Covetrus, 1XYL006) to maintain controlled respiration. Euthanasia was conducted on 2, 4, and 6 dpi (*n* = 4 pigs per time point per condition) using the higher anesthetic dose followed by sodium pentobarbital (Euthapen, Euthsol-100) according to approved veterinary procedures.

#### Recipient animals and airborne exposure

To assess airborne transmission, naïve sentinel pigs (*n* = 3 per environmental condition) were moved 24 h post-inoculation and housed 4 m from the nearest donor pens within the same room. There was no shared equipment, no personnel crossover during daily sampling, and no direct or indirect contact between donor and recipient pigs; air served as the sole transmission interface. Recipients were monitored from 1 to 6 days post-exposure (dpe) in parallel with donor surveillance.

#### Breath aerosol collection

Breath aerosols were collected from anesthetized donor pigs using the PathoSift Pro sampler. Animals were positioned with nose aligned directly to the conical inlet without direct contact with the sampler (~5 cm from the inlet), and exhaled particles were collected for 30 min into chilled Opti-MEM supplemented with penicillin/streptomycin (100 μg/mL) (R15140-122; Life Technologies Corporation). Collection reservoirs were maintained on ice, and samples were aliquoted immediately after acquisition and stored at −80°C before further analyses.

#### Environmental air sampling

Airborne virus was collected using the PathoSift Pro system positioned at three spatial locations relative to each donor pen: (i) immediately adjacent to the pen boundary, (ii) in the hallway approximately 0.5 m from the pen, and (iii) in the adjacent pen, also adjacent to the vent of the donor animal pen. Air was sampled for 60 min per time point under each T/RH condition, with media maintained on ice throughout collection to preserve viral infectivity.

#### Nasal washes

Nasal washes were obtained daily from both donor pigs (0–6 dpi) and sentinel pigs (1–6 dpe). A total of 3 mL PBS containing penicillin/streptomycin was instilled into each nare and recovered by gravity drainage into sterile collection tubes.

#### Lung lavage

Bronchoalveolar lavage fluid (BALF) was collected right before necropsy (2, 4, and 6 dpi) by instilling 10 mL PBS into the trachea and aspirating the returned fluid. Samples were immediately placed on ice and processed for downstream virological assessment. Whole blood was collected from donor pigs before delivering and on 0 dpi via venipuncture. Serum was tested to confirm seronegativity using HAI against the challenge virus as well as the contemporary seasonal subtype H1N1 virus, A/Missouri/CS20N08/2020 (H1N1), prior to challenge.

#### Transmission confirmation

Airborne transmission was confirmed by recovery of infectious virus or RNA in nasal washes of sentinel pigs together with concurrent detection of virus in donor breath aerosols and environmental air samples within each T/RH condition. The absence of shared air handling and physical separation precluded alternative transmission routes and ensured airborne-only exposure.

### Particle size distribution measurement

Particle size distribution was quantified using an Aerodynamic Particle Sizer (APS Model 3321; TSI). For each measurement, pigs were anesthetized and fitted with a customized polylactic acid (PLA) pig mask sealed with a rubber gasket (Fig. 2a). The mask contained a one-way valve to supply fresh air to the animal and a second port connected to the APS via a Tygon tube to stabilize sampling conditions and minimize particle losses. APS data were acquired for 5 min using Aerosol Instrument Manager software (Version 10.3, TSI) and exported to a TXT file for downstream analysis. Measurements for each animal were performed in triplicate.

To account for ambient aerosol contributions, background particle concentrations in the experimental environment were measured using the APS prior to each sampling session under the same conditions (Fig. S2). These background measurements were incorporated into the data analysis by subtracting the number of background particles from the particles measured by APS to ensure that the reported particle concentrations reflected pig-derived emissions rather than ambient aerosol levels.

### Aerosol transport and fate experiments

To evaluate how environmental conditions influence influenza persistence independent of host emission, two T/RH conditions were tested, including 7°C/73% RH and 20°C/50% RH. CA/04-H1N1 was propagated in MDCK-SIAT1 cells and titrated by TCID_50_. Viruses were diluted in Opti-MEM I (11058-021 reduced serum medium; Life Technologies Corporation) supplemented with penicillin/streptomycin (15140-122; Life Technologies Corporation) at 100 μg/mL to 6 × 10^6^ TCID_50_ in 40 mL and aerosolized using a six-jet Collison nebulizer operating at 30 psi. For each condition, samples were collected at five distances (0.25, 0.5, 1, 2, and 3 m) from the aerosol source using the PathoSift Pro sampler, which enables size-segregated collection of ≤5 and >5 µm. Each experiment was performed in triplicate. Generalized additive models (GAMs) were used to evaluate the relationships among T/RH, distance, and viral outcomes (TCID_50_ and RNA copies).

### Statistical analysis

Statistical analyses were performed using GraphPad Prism version 10.2.0 (GraphPad Software, Boston, MA, USA) and R (version 2025.09.2, 418). Longitudinal nasal wash and breath measurements were analyzed using linear mixed-effects models with T/RH condition and day post-infection as fixed effects and animal as a random effect. Recipient (sentinel) nasal wash viral RNA and infectious titers across days post-exposure were analyzed using two-way repeated-measures ANOVA with T/RH condition and day post-exposure as factors; Tukey’s test was used for multiple comparisons (*α* = 0.05). Aerosolization experiments were analyzed using generalized additive models (GAMs) to evaluate the effects of distance and T/RH condition (including their interaction) on viral RNA and infectivity outcomes. *P* values <0.05 were considered statistically significant.

## Data Availability

All data supporting the findings of this study are available within the article and its supplementary materials. For additional information, please contact the corresponding author.

## SUPPLEMENTAL MATERIAL

Supplemental material jvi.00634-26-s0001.pdf Fig. S1 to S3.

ASM does not own the copyrights to Supplemental Material that may be linked to, or accessed through, an article. The authors have granted ASM a non-exclusive, world-wide license to publish the Supplemental Material files. Please contact the corresponding author directly for reuse.
