# Supplementary material for: Environmental temperature and relative humidity shape post-emission aerosol fate and airborne influenza transmission
Source: J Virol. 2026 Jun 15;100(7):e00634-26. doi: 10.1128/jvi.00634-26 (PMC13386918; doi:10.1128/jvi.00634-26)
Supplement: Supplemental material — Fig. S1 to S3. [file jvi.00634-26-s0001.pdf]

Supplementary Information

Title: Environmental temperature and relative humidity shape post-emission aerosol fate and airborne influenza transmission

Running title: Temperature and humidity shape airborne influenza transmission

Author list

Xuan-Dung Nguyen[a,b,c,d], Bac Tran Le[a,b,d], Jacob Bleich[a,b,d], Constanza Espada[a,b,d], Wei Zhang[a,b,d], and Xiu-Feng Wan[a,b,c,d]*

Affiliations

[a]NextGen Center for Influenza and Emerging Diseases, University of Missouri, Columbia, MO 65211, USA; [b]Department of Molecular Microbiology and Immunology, School of Medicine, University of Missouri, Columbia, MO 65211, USA; [c]Department of Electrical Engineering & Computer Science, College of Engineering, University of Missouri, Columbia, MO 65211, USA; [d]Bond Life Sciences Center, University of Missouri, Columbia, MO 65211, USA

*Correspondence: wanx@missouri.edu

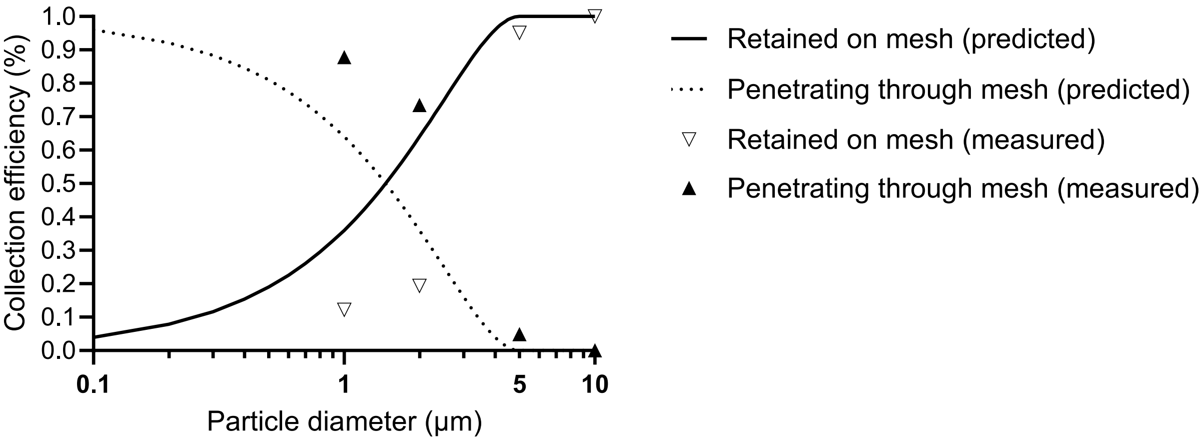

FIG. S1. Validation of particle transmission through the 5-μm stainless steel mesh used in the PathoSift Pro sampler. Fluorescent polystyrene latex (PSL) particles with sizes of 1, 2, 5, and 10 μm diameter were aerosolized and sampled using the PathoSift Pro under the same flow conditions used in the animal transmission experiments. Particle collection efficiency was quantified in the mesh retained fraction and the downstream BioSampler collection fraction. Particles larger than the mesh opening were fully retained under the tested conditions, whereas most of 1-μm and 2-μm particles were transmitted through the mesh and recovered downstream.

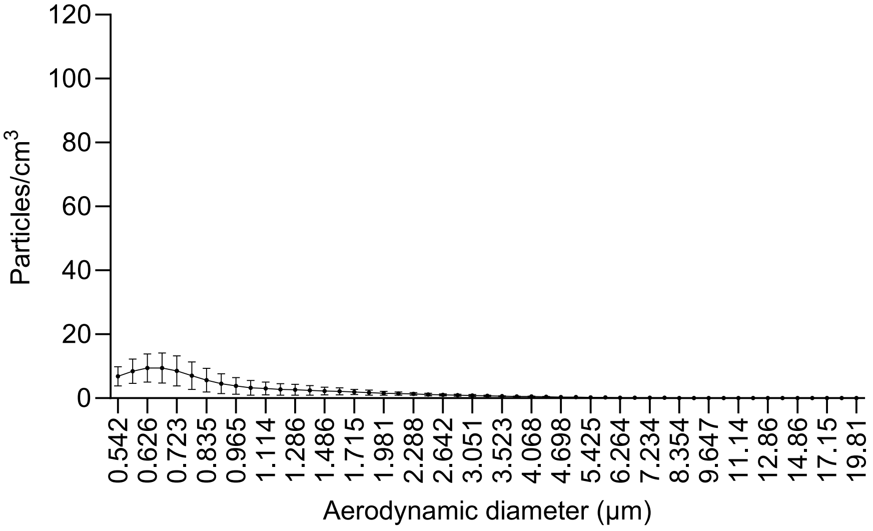

FIG. S2. Background aerosol particle size distribution. Background aerosol particle size distributions in the experimental environment measured using the APS prior to pig sampling. Background measurements were collected in triplicate to quantify ambient particle levels during particle emission experiments. Data are shown as mean ± standard deviation (SD).

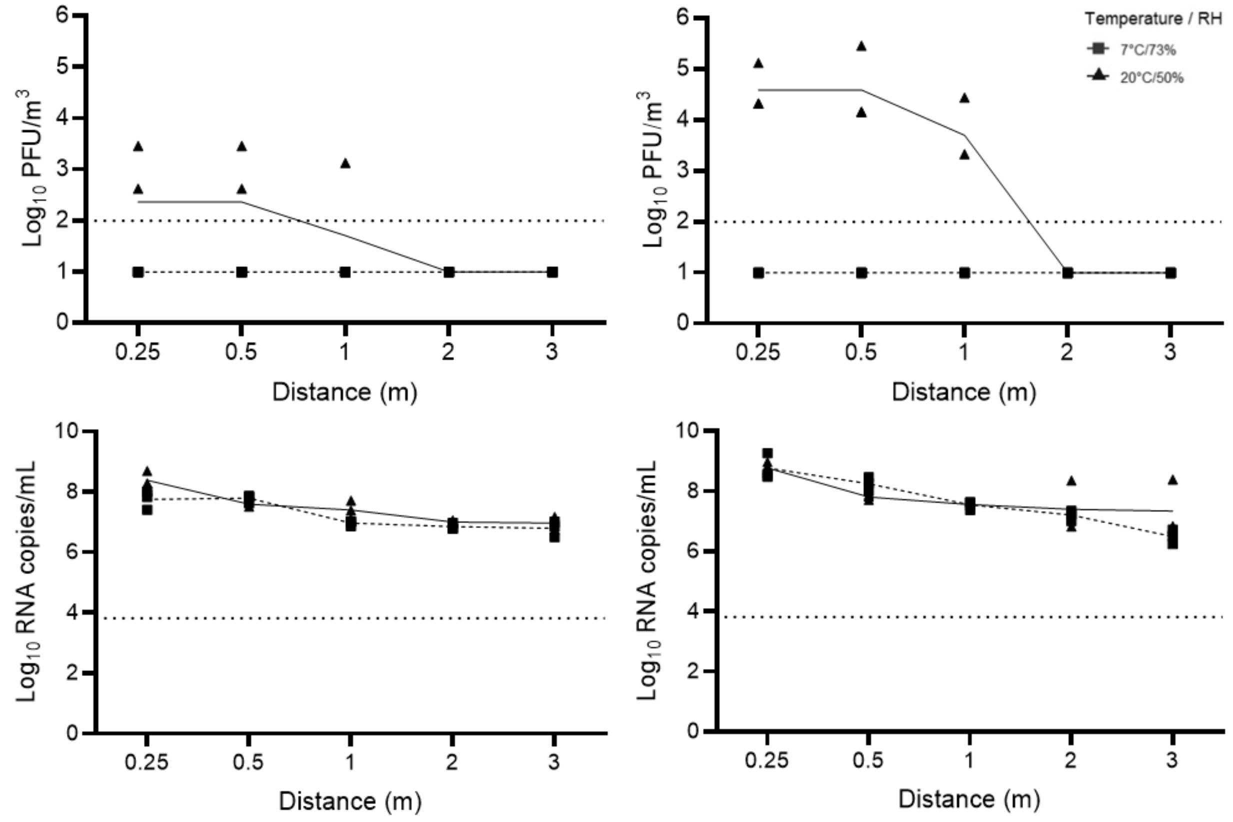

FIG. S3. Environmental temperature–humidity conditions influence distance-dependent recovery of airborne influenza virus during aerosol transport. CA/04-H1N1 virus was aerosolized and sampled at increasing distances (0.25, 0.5, 1, 2, and 3 m) under two environmental conditions: 20 °C/50% RH (ambient) and 7 °C/73% RH (cold/high humidity). Size-segregated virus-laden particles were collected using PathoSift Pro to distinguish aerosols (≤ 5 µm) from droplets (> 5 µm). Distance-dependent recovery of infectious virus (PFU/m^3) and viral RNA levels (log_{10} RNA copies/m^3) is shown for aerosols (≤ 5 µm) and droplets (> 5 µm) under each T/RH condition. Experiments were performed in triplicate. Each point represents a single measurement, and lines represent the mean across replicates at each distance.
